# Can Stochastic Sensing of Amino Acids in Whole Blood, Urine, and Saliva Predict Gastric Tumor Growth and Extension?

**DOI:** 10.3390/life16101689

**Published:** 2026-10-09

**Authors:** Alexandra Orășeanu, Raluca-Ioana Stefan-van Staden, Ramona Georgescu-State, Ionela Raluca Comnea-Stancu, Catalina Cioates Negut, Bianca-Maria Tuchiu-Stanca, Damaris-Cristina Gheorghe, Ruxandra-Maria Ilie-Mihai, Andrada-Ioana Stefan, Jacobus (Koos) Frederick van Staden, Ciprian Brisc, Alexandru Adrian Bratei

**Affiliations:** 1 Doctoral School of Biomedical Sciences, University of Oradea, 410087 Oradea, Romania; adaoraseanu@yahoo.com (A.O.); brisciprian@gmail.com (C.B.); 2 Clinic of Gastroenterology, Bihor Clinical County Emergency Hospital, 410169 Oradea, Romania; 3 Laboratory of Electrochemistry and PATLAB, National Institute of Research and Development for Electrochemistry and Condensed Matter, 202 Splaiul Independentei, 060021 Bucharest, Romania; state_ramona@yahoo.ro (R.G.-S.); negutcatalina79@gmail.com (C.C.N.); gheorghe.damaris16@gmail.com (D.-C.G.); i.ruxandra04@yahoo.com (R.-M.I.-M.); andradastefan311@gmail.com (A.-I.S.); koosvanstaden2012@gmail.com (J.F.v.S.); 4 Clinical Nephrology Hospital “Carol Davila”, 4 Grivitei Street, 010731 Bucharest, Romania

**Keywords:** amino acids, gastric cancer, enantiomers

## Abstract

**Background**: Although amino acids have been correlated with gastric cancer progression, most previous investigations have focused on a single biological matrix and have not differentiated between the two enantiomeric forms. **Methods**: In this study, we investigated the associations between the stereoisomeric profiles of 12 amino acids in whole blood, urine, and saliva and the clinicopathological features of gastric cancer. Samples from 33 patients were analyzed using a stochastic sensing platform, and the data were processed with Excel and MATLAB R1. **Results**: Distinct amino acid patterns were associated with tumor location, size, thickness, local extension, and pathological T stage. Specific stereoisomeric profiles were associated with tumors extending to the pylorus, duodenum, and esophagus, as well as with disease stage. **Conclusions**: These findings suggest that a combined analysis of L- and D-amino acid stereoisomers across multiple biological fluids may yield useful minimally invasive molecular profiles for monitoring gastric cancer progression and invasion.

## 1. Introduction

Tumor cell metabolism has become of interest in recent years with the development of biomolecule detection methods and the establishment of several metabolic pathways [1,2].

Among the most important biomolecules for metabolism are amino acids, which are indispensable for the biosynthesis of proteins involved in the metabolic pathways of both normal and tumor cells. Monitoring changes in the amino acid profile targets metabolic reprogramming as an important component of oncogenic programs [3,4,5,6,7,8].

Cancer cells require adequate levels of proteinogenic amino acids to proliferate and survive. Temporarily replacing a normal diet with an artificial one that selectively modifies amino acid levels or manipulating amino acid levels in the tumor microenvironment could potentially hinder cancer cell growth and survival [9,10].

Initially, only L-amino acids were considered important in metabolism until 1939, when Kögl et al. highlighted the presence of D-Glu [11] in tumor cells, which opened the door to evaluating their presence in other eukaryotic cells [12,13,14,15,16,17,18,19].

Given the important role of amino acids in the development of the neoplastic process, monitoring the molecular amino acid profile has the potential to provide highly useful information in this regard. To this end, we decided to investigate aspects related to the growth, extent, and invasiveness of gastric carcinomas in relation to the amino acid profile determined using stochastic sensors.

In this study, we used a stochastic sensor to investigate the associations between the levels of 12 amino acids—glutamine (Gln), tryptophan (Trp), cysteine (Cys), methionine (Met), lysine (Lys), alanine (Ala), phenylalanine (Phe), valine (Val), serine (Ser), leucine (Leu), ornithine (Orn), and pipecolic acid (Pip)—both L- and D-stereoisomers, in biological fluids (whole blood, urine, and saliva) and the growth and extension of tumors in gastric cancer patients.

## 2. Materials and Methods

### 2.1. Stochastic Sensing Platform Design

**Figure 1:**
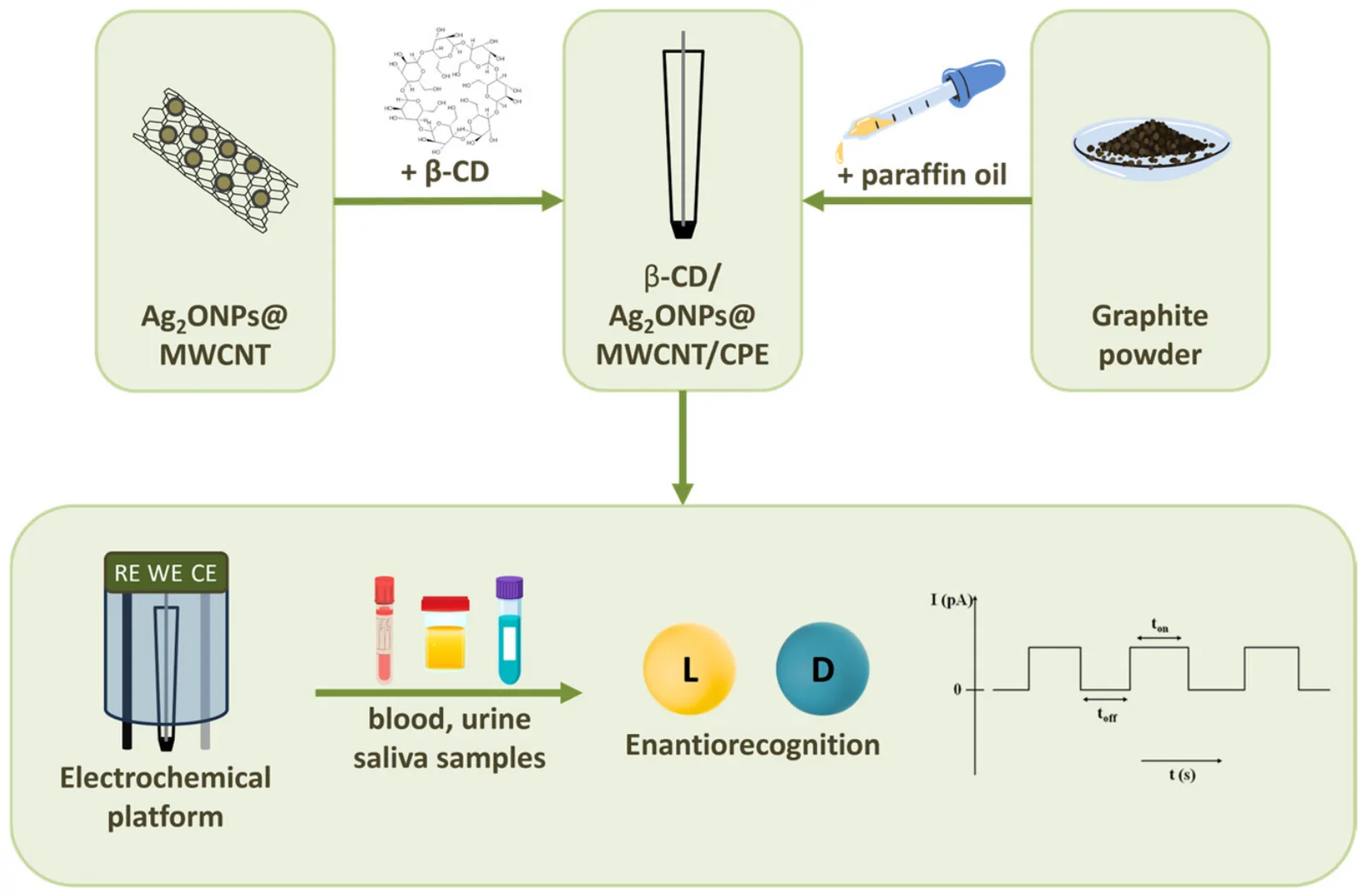
Fabrication of the stochastic sensor, integration of it in the platform,, and stochastic sensing principle.

The stochastic sensing platform comprised a β-cyclodextrin/silver oxide nanoparticles-decorated multi-walled carbon nanotube composite (β-CD/Ag2ONPs@MWCNT/CPE) as the working electrode, an Ag/AgCl pseudo-reference electrode, and a Pt counter electrode. The preparation of the working electrode followed our previously reported protocol, beginning with the synthesis of Ag_2_ONPs using green tea extract, followed by the preparation of the Ag_2_ONPs@MWCNT composite and functionalization with β-cyclodextrin to ensure selective molecular recognition. Figure 1 summarizes the fabrication workflow and the configuration of the sensing platform. Electrochemical stochastic measurements were acquired using an EmStat Pico portable potentiostat (PalmSens, Houten, The Netherlands) connected to the assembled sensing platform [20].

### 2.2. Stochastic Sensing Approach

Stochastic measurements were carried out at a constant applied potential using the previously developed β-CD/Ag_2_ONPs@MWCNT/CPE sensing platform. The stochastic signal is generated from two consecutive events. First, the analyte transiently obstructs the conductive channel, resulting in the t_off_ value, which serves as its molecular signature and enables qualitative identification according to molecular size, shape, and stereochemical configuration. Next, the analyte interacts with the channel wall after entering the sensing site. The corresponding t_on_ value depends on the analyte concentration and is used for quantitative determination.

Amino acid enantiomers in biological samples were identified by matching their t_off_ values with those obtained during calibration. Their concentrations were then calculated from the calibration relationship, 1/t_on_ = a + b × concentration, using the measured t_on_ values. Both t_off_ and t_on_ values were obtained from chronoamperometric recordings, while calibration equations were established by linear regression.

**Figure 2:**
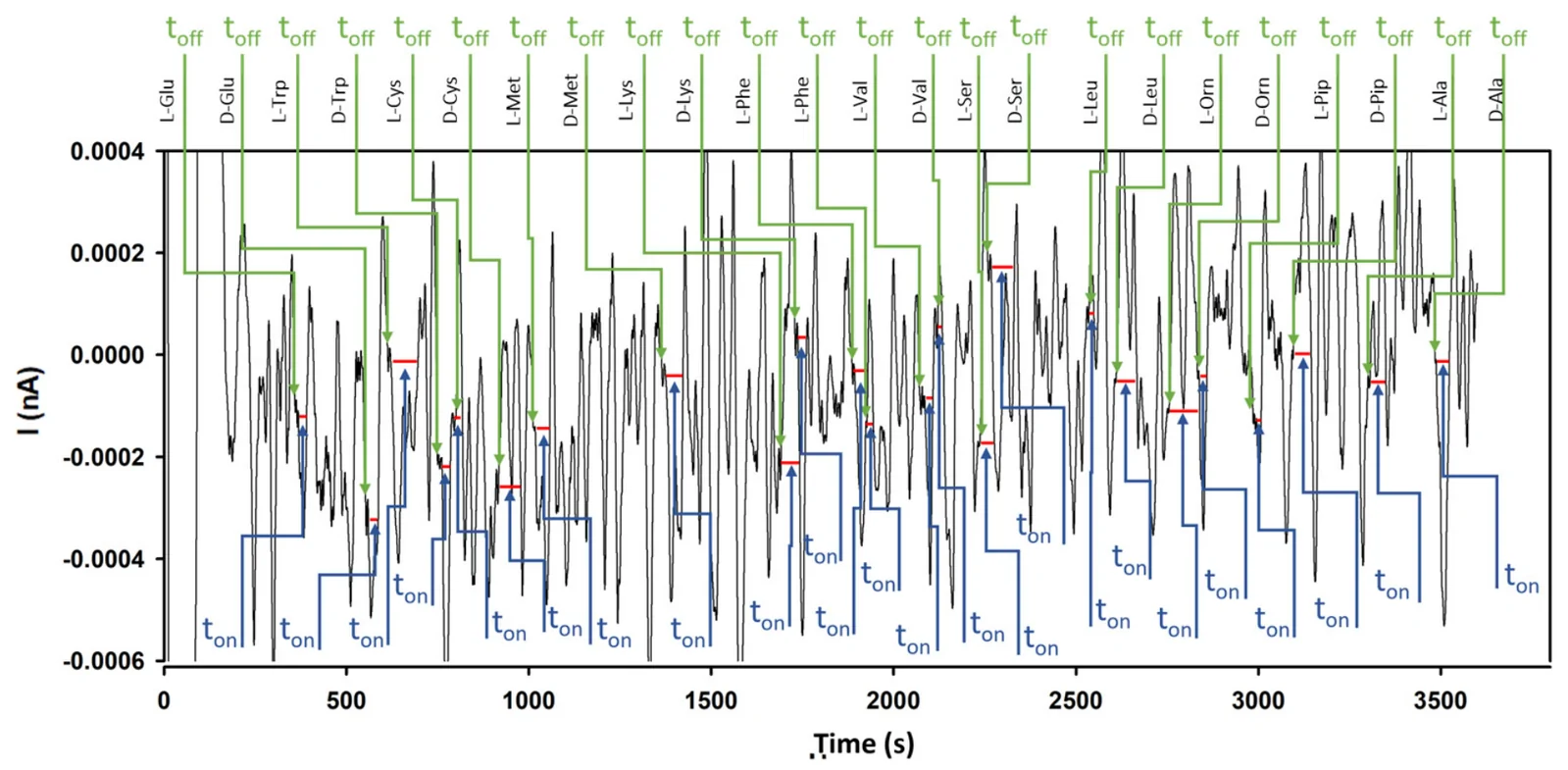
Representative stochastic response recorded in a whole blood sample using the β-CD/Ag_2_ONPs@MWCNT/CPE sensing platform.

Figure 2 exhibits a representative stochastic response recorded in a whole blood sample using the proposed sensing platform.

### 2.3. Biological Samples

The proposed stochastic sensing platform was employed to simultaneously detect and quantify the L- and D-enantiomers of 12 amino acids in whole blood, saliva, and urine collected from 33 patients with gastric cancer. Their characteristics are described in Table 1. The study was conducted following protocol approval granted by the County Emergency Hospital from Târgu-Mureș (Approval nr. 32647/2018) and the Ethics Committee of the County Emergency Clinical Hospital Bihor (Approval No. 35965/21.11.2024). Written informed consent was obtained from every participant before sample collection.

The concentrations of each of the 24 amino acids measured in the three biological fluids, yielding a total of 72 variables, were evaluated for associations with the presence or absence of invasion or metastasis.

To identify the amino acids included in the subsequent analyses, 72 comparisons were performed for each clinicopathological parameter. Accordingly, after applying the Bonferroni correction, the significance threshold was set at α = 0.000694, corresponding to a family-wise error rate (FWER) of 4.87%.

Following each comparison, cut-off thresholds of 10%, 20%, or 30% were applied to assess the presence of each amino acid in relation to the selected clinicopathological parameter. These thresholds were chosen because of the limited number of patients included in the study. Consequently, the present investigation should be regarded primarily as hypothesis-generating rather than confirmatory.

After amino acid selection, the number of amino acids meeting the predefined criteria was calculated. Logistic regression analysis was subsequently performed to estimate *p*-values and evaluate the associations between the number of selected amino acids and the selected parameters.

## 3. Results

In this study, it was observed how the levels of selected amino acids vary in both L and D stereoisomeric forms. First, it was observed whether there was any relationship between the selected morphopathological parameters and the presence or absence of an amino acid, and then, where appropriate, it was investigated further by comparing the values of the selected parameters with those of the amino acids in the 3 biological fluids—whole blood, urine, and saliva.

For this study, stochastic sensors were used that have a very low detection limit and offer numerous advantages. Thus, amino acids that present levels below the detection limit in the samples taken from biological fluids were considered undetectable.

To evaluate the growth and extension of the tumor, the chosen histopathological parameters are the maximum dimension that evaluates the growth on the surface, the maximum thickness that evaluates the growth in the depth of the organ, the extensions towards the gastric cardia and pylorus that evaluate the transition from the stomach to the esophagus and the transition from the stomach to the duodenum, and the pT parameter from the TNM staging system that evaluates both the growth in depth and the capacity to invade the layers of the gastric wall.

Before analyzing these parameters, it was investigated whether there was any relationship between the levels of amino acids in the three biological fluids and the location of the tumor. For this purpose, the tumors were divided into five categories: subgastric, cardial and fornix, fornix and gastric body, gastric body, gastric body and antrum, and antrum and/or pylorus.

The evaluation of the position was obtained by highlighting the presence or absence of amino acids in the selected biological fluids. Thus, the following aspects were observed:

○ A tumor located at the level of the gastric fornix and gastric cardia was related to levels of L-Ser and D-Leu in whole blood and to levels of D-Trp, D-Cys, and L-Val in saliva;
○ A tumor located at the level of the body was correlated with levels of L-Met and D-Phe in whole blood, and with levels of D-Phe and D-Ser in saliva;
○ A tumor located at the level of the antrum and pylorus was related to levels of L-Glu, L-Cys, D-Met, L-Ala, L-Phe, L-Pip, and D-Pip in whole blood and to L-Val in urine.

**Figure 3:**
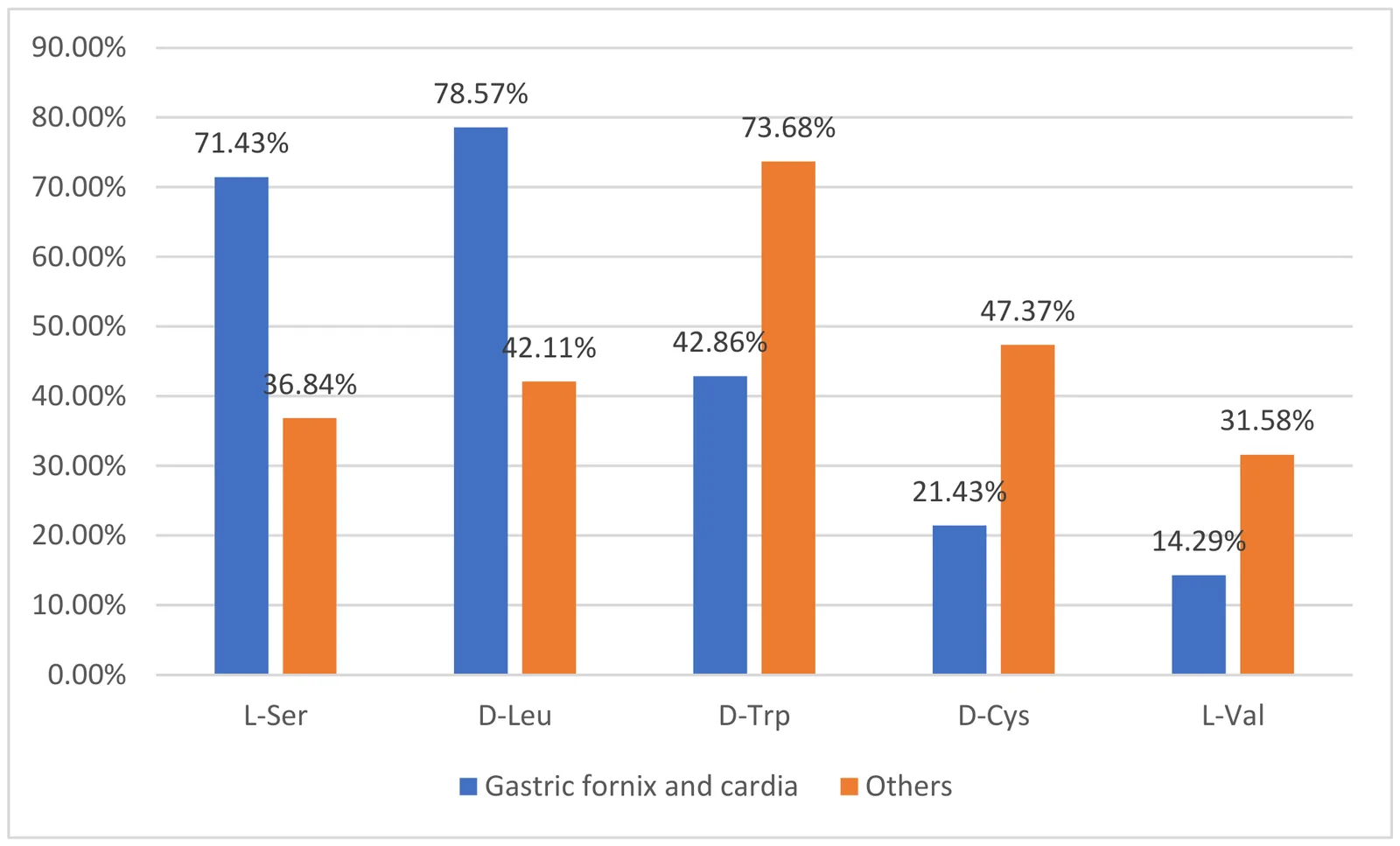
Percentages of patients with detectable levels of selected amino acids by tumor location in gastric fornix and cardia vs. other locations.

For tumors located at the level of the fornix and gastric cardia, it was noted that they are mainly associated with the presence of L-Ser and D-Leu in whole blood and the absence of D-Trp, D-Cys, and L-Val in saliva. The percentage data are shown in Figure 3.

By counting the amino acids with detectable and undetectable levels among the selected amino acids, the following was noted:

○ By counting the positively correlated amino acids (L-Ser and D-Leu), it was observed that 85.71% of those with tumors located in the fornix or gastric cardia present at least one amino acid with detectable levels, compared to only 61.11% of those with other locations;
○ By counting the negatively correlated amino acids (D-Trp, D-Cys, and L-Val), it was observed that only 64.29% of those with tumors located in the fornix or gastric cardia present at least one amino acid with detectable levels, compared to only 94.77% of those with other locations.

The percentages above are not that significant, but if we differentiate between the number of amino acids positively correlated with detectable levels and the number of amino acids negatively correlated with detectable levels, we obtain that 78.57% of those with tumors located in the fornix or gastric cardia present a positive difference value compared to only 19.05% of those with other tumor locations.

**Figure 4:**
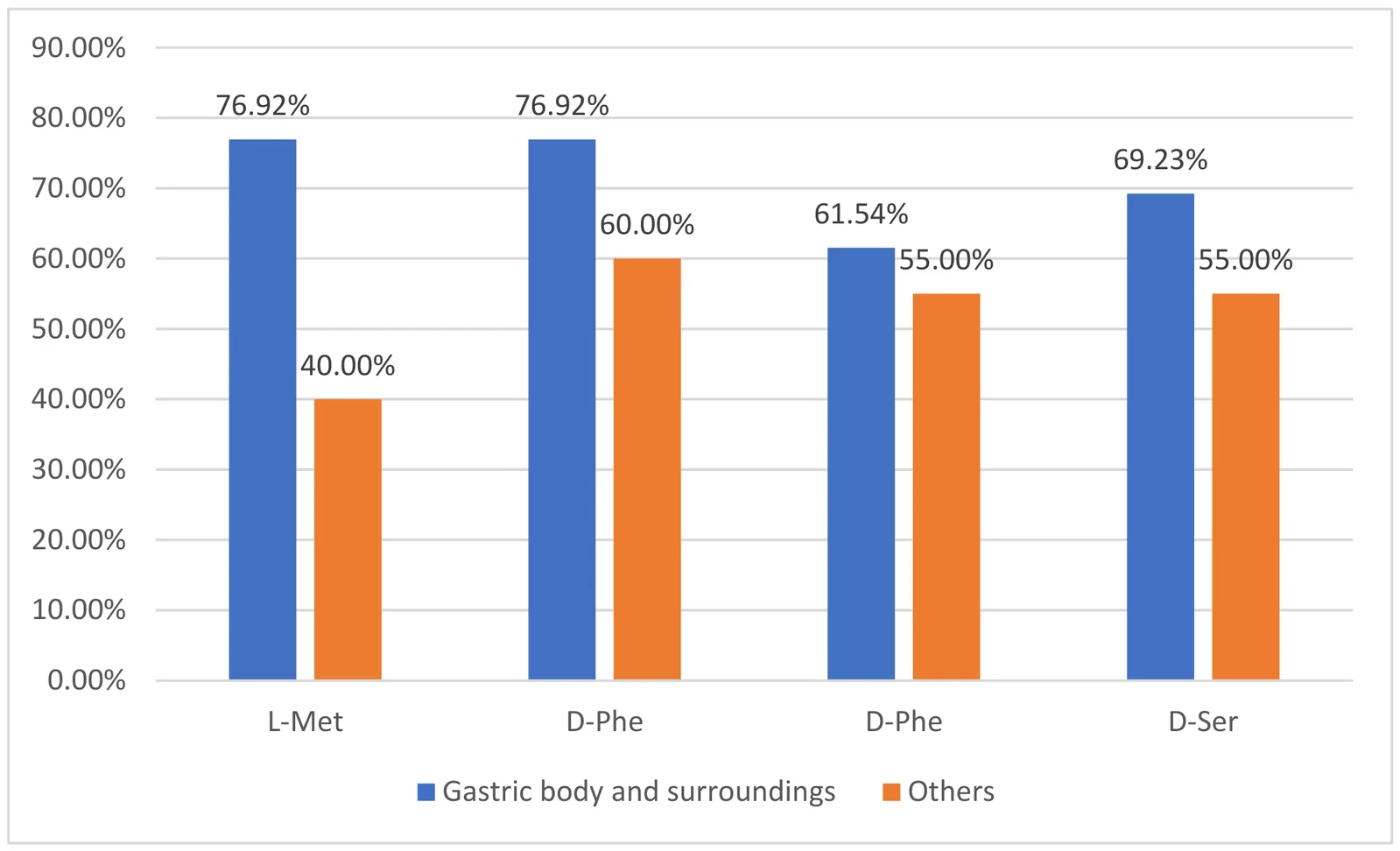
Percentages of patients with detectable levels of selected amino acids by tumor location in gastric body and surroundings vs. other locations.

Tumors located in the gastric body were positively correlated with L-Met and D-Phe levels in whole blood, and with D-Phe and D-Ser levels in saliva. These can be seen in Figure 4.

By counting the amino acids with detectable levels among the selected amino acids, it was observed that all patients with tumors located in the gastric body had at least three amino acids with detectable levels, compared to only 34.61% of the remaining patients.

**Figure 5:**
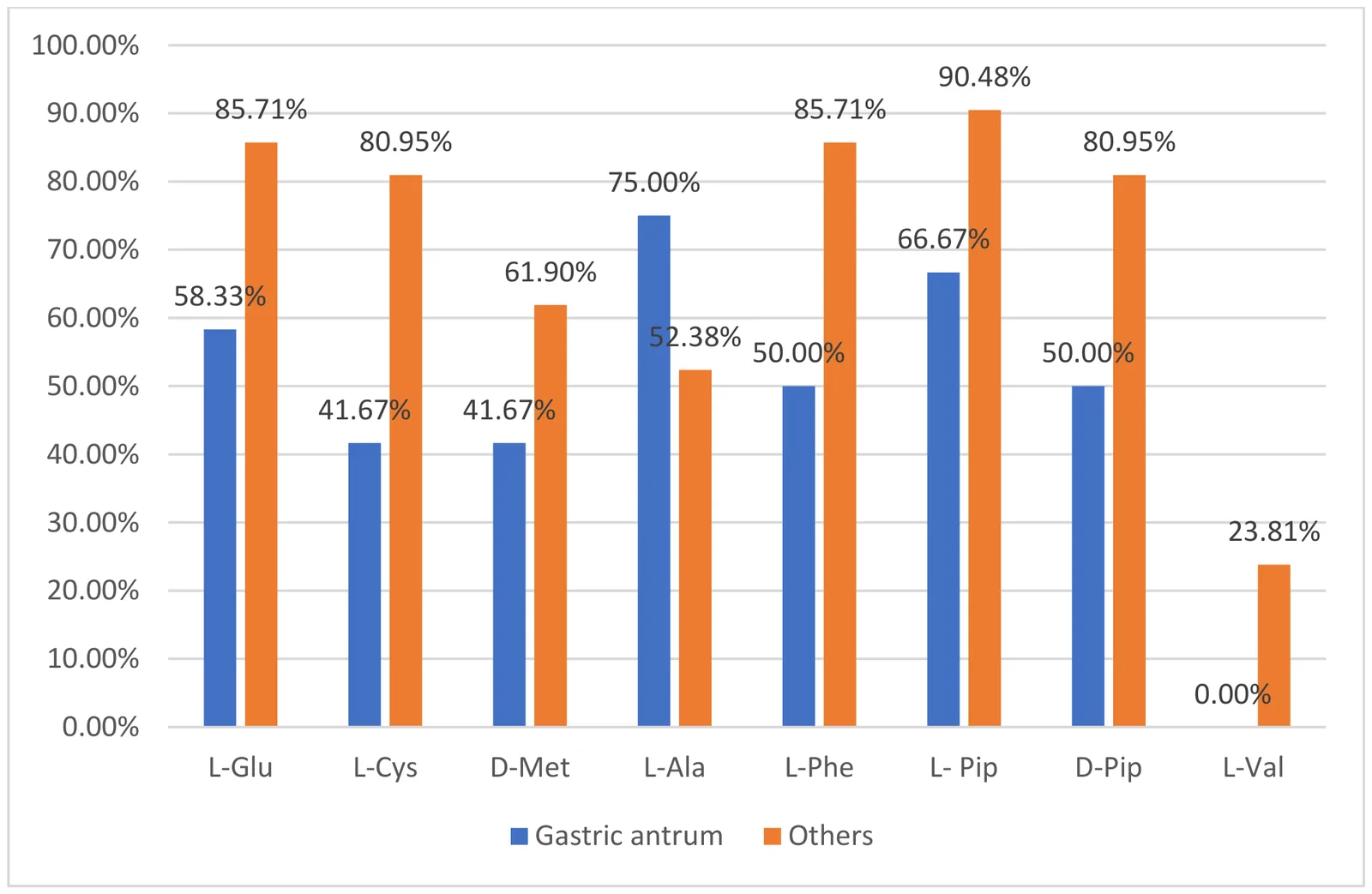
Percentages of patients with detectable levels of selected amino acids by tumor location in gastric antrum vs. other locations.

For the last location, namely at the level of the gastric antrum and pylorus, this was negatively correlated with the presence of L-Glu in whole blood, L-Cys in whole blood, D-Met in whole blood, L-Phe in whole blood, D-Pip in whole blood, and L-Val in urine and positively correlated with the presence of L-Ala in whole blood according to Figure 5.

If we differentiate between the number of negatively correlated and positively correlated amino acids, it was observed that 75% of patients with tumors in the antrum present at most four amino acids with detectable levels, while 71.43% of those with other tumor locations present at least five amino acids with detectable levels.

Now that the molecular profile of the stochastically determined amino acids was analyzed, the tumor dimensions were analyzed.

The first dimension analyzed is the maximum length on the surface. To highlight the relationships between the molecular profile of the stochastically detected amino acids and this parameter, the tendency of decrease or increase in the parameter value with the level of each detectable amino acid was followed. Table 2 was thus created.

**Figure 6:**
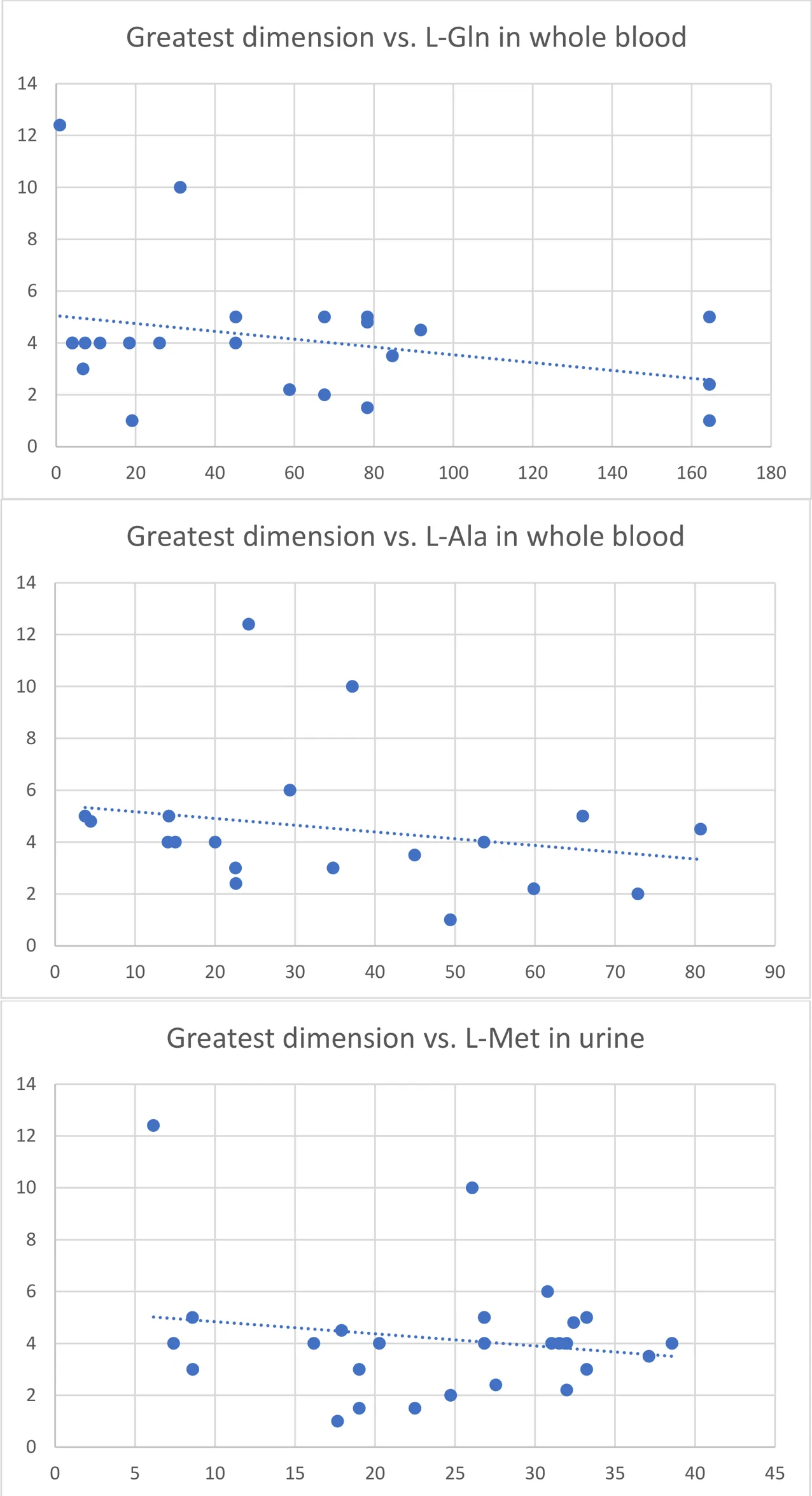

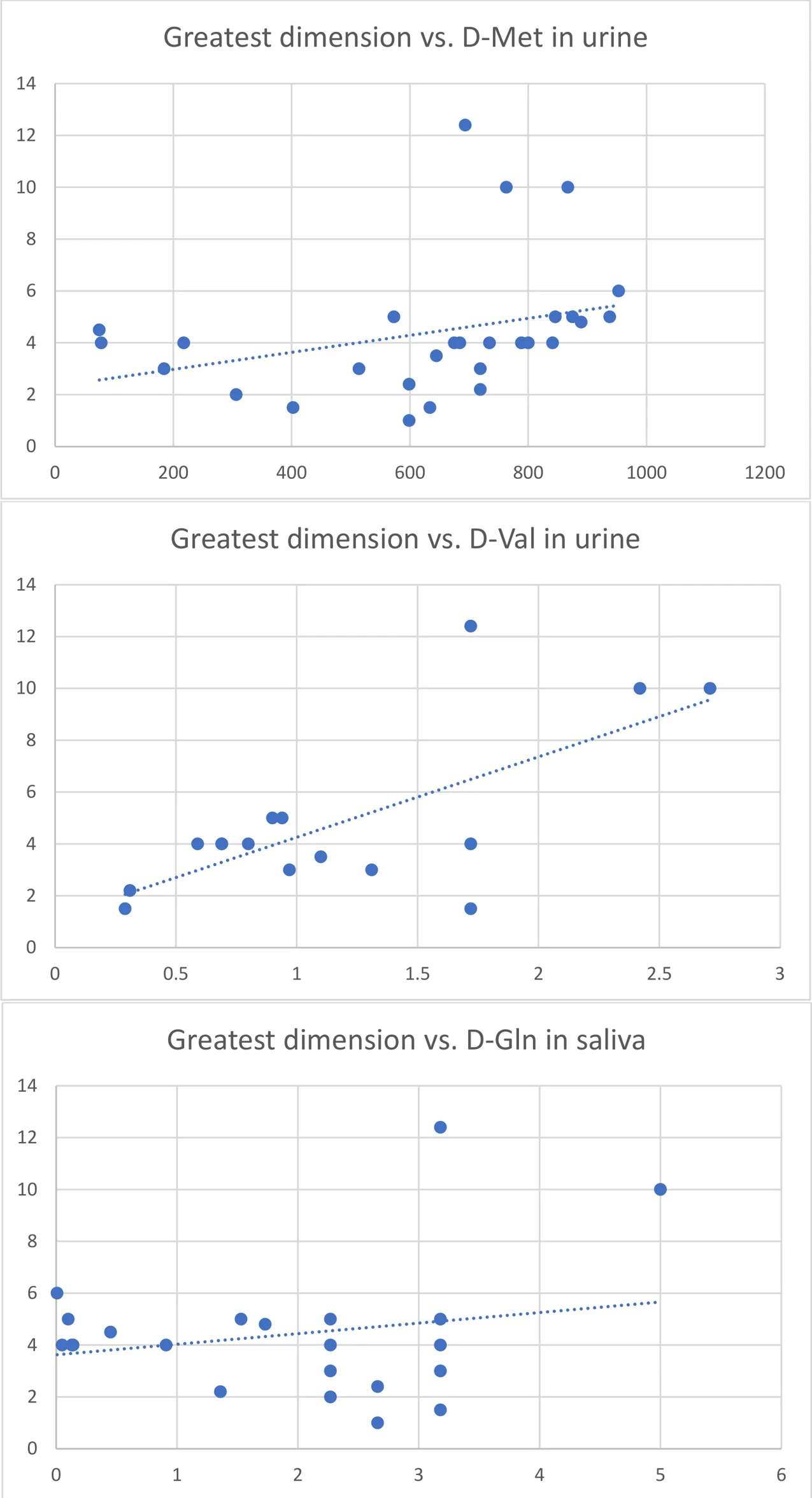

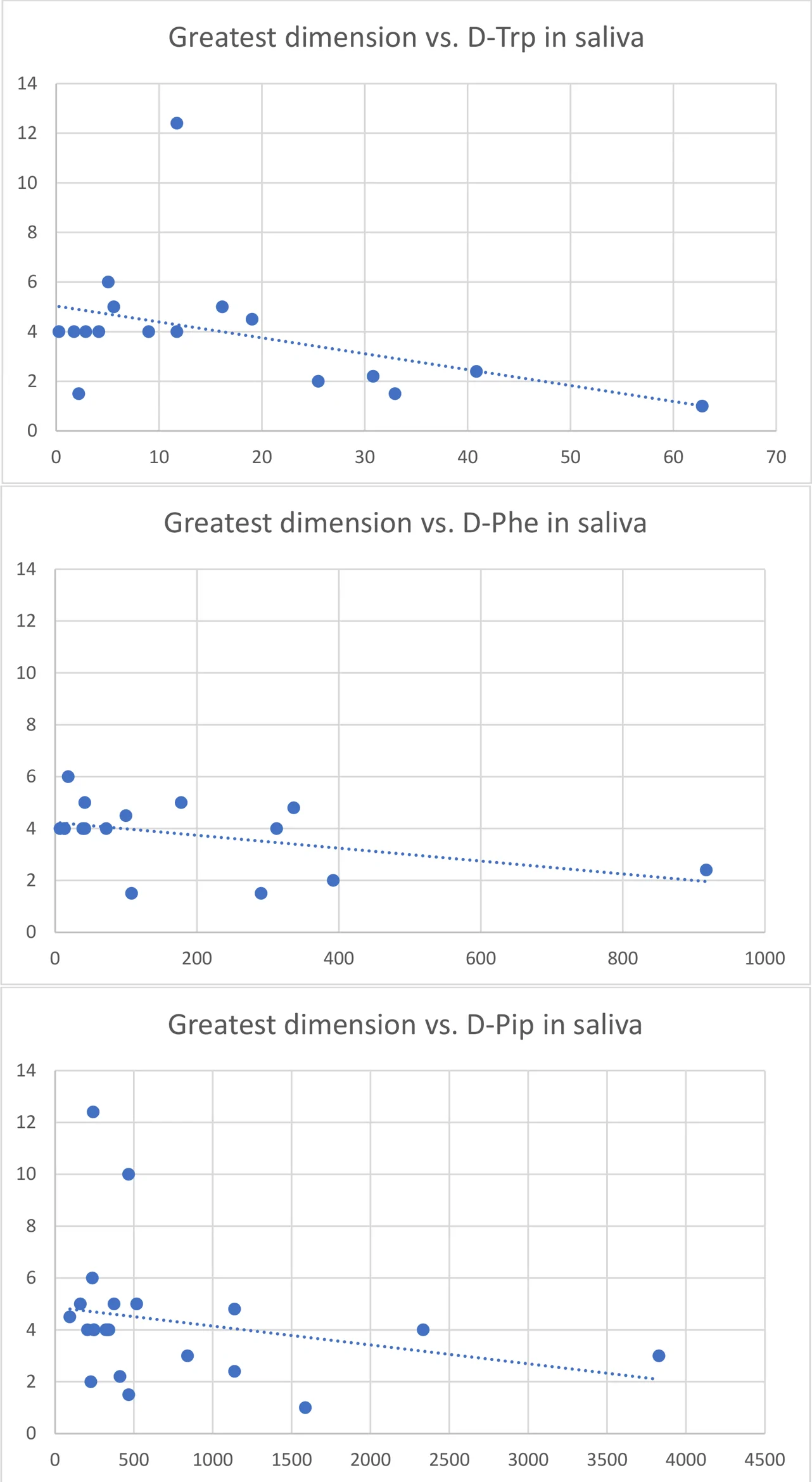
Graphical representation of maximum dimension in surface growth/[cm] as a function of the levels of representative amino acids in each biological fluid.

To evaluate how amino acid levels evolve with the value of this parameter, the following graphs were created and included in Figure 6.

From the analysis of the graphs in Figure 4, it can be seen that a larger size is associated with a decrease in the concentrations of L-Gln in whole blood, L-Ala in whole blood, L-Met in urine, D-Trp in saliva, D-Phe in saliva, and D-Pip in saliva, with an increase in the concentrations of D-Met in urine, D-Val in urine, and D-Gln in saliva.

The next parameter analyzed is the maximum thickness (width). For this parameter, the procedure was similar to that for the maximum dimension in surface growth. The following table was thus created (Table 3).

**Figure 7:**
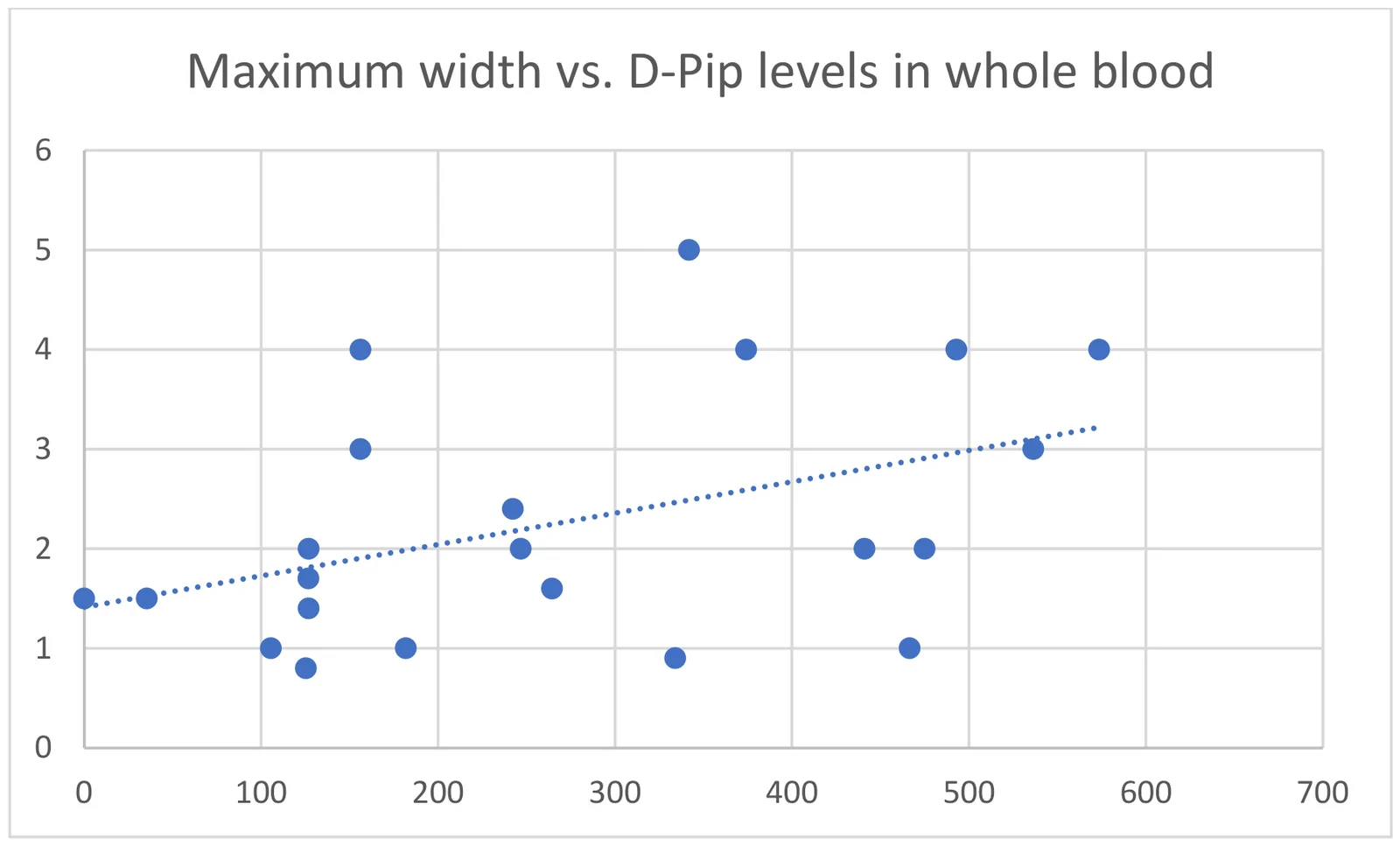

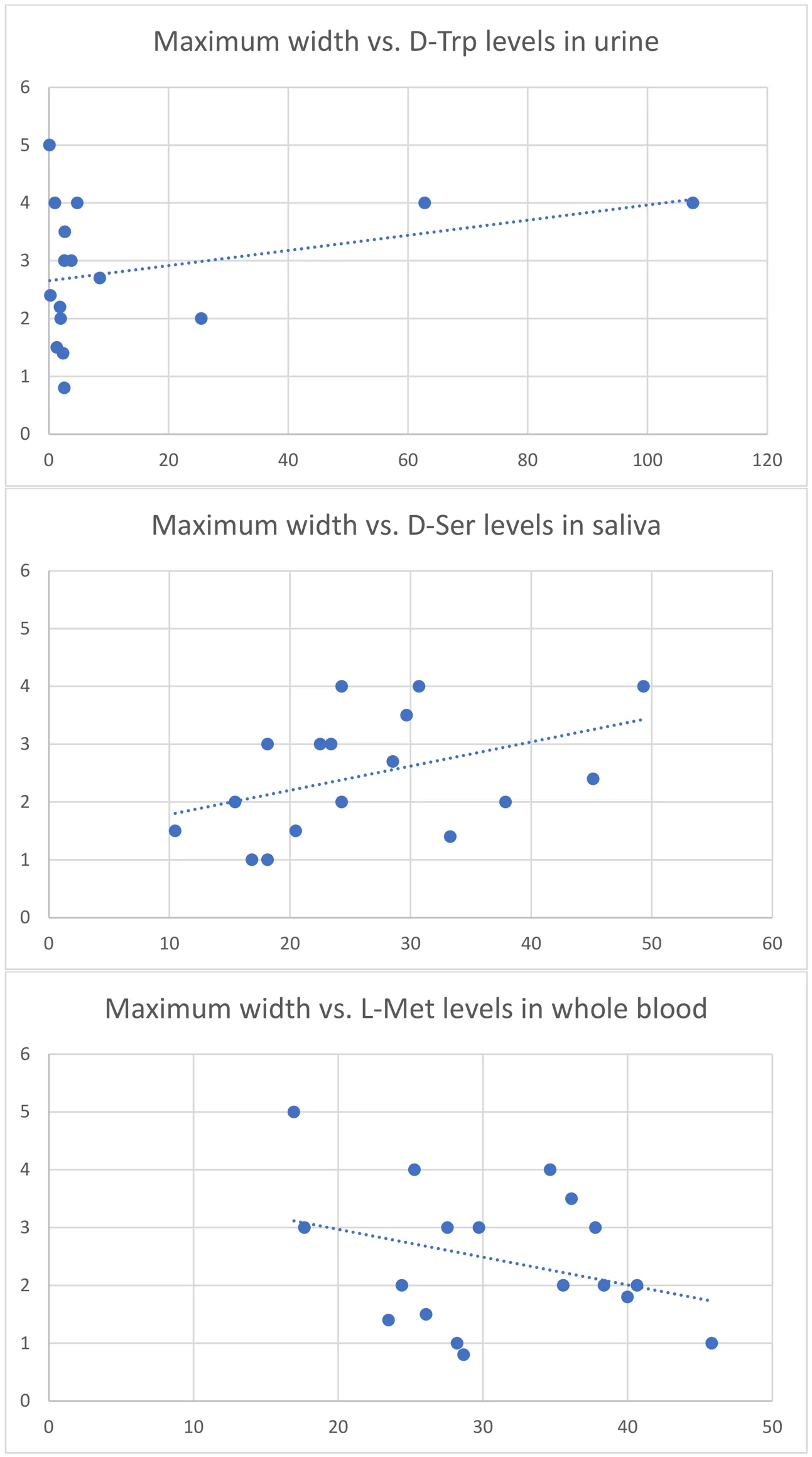

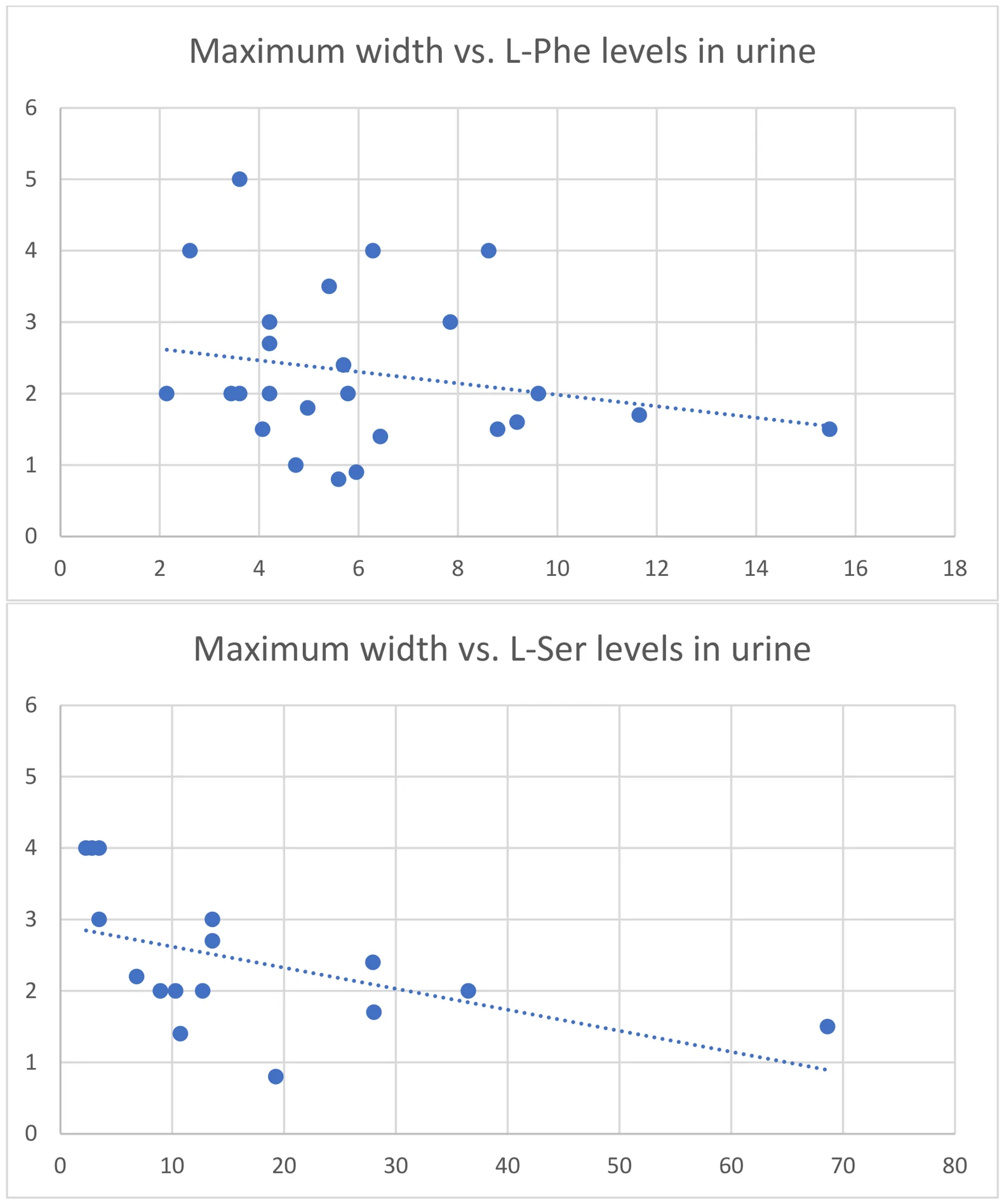
Graphical representation of maximum width/[cm] as a function of the levels of representative amino acids in each biological fluid.

Main plots regarding the correlations between the width of the tumor and the most representative amino acids are given in Figure 7.

So far, the main tumor dimensions that characterize the growth of the tumor process have been evaluated. The tumor thickness can be linked to the invasive nature in depth, and further, the pT parameter from the TNM staging will be analyzed to complete the information provided by the tumor thickness. The maximum size is associated with growth in the surface and the ability of the tumor process to extend within the stomach. Further, the evolution of the molecular profile in stochastically detected amino acids will be analyzed when the tumor extends to the gastric cardia and esophagus and to the pylorus and duodenum.

Extension to the pylorus will be discussed first. In the database, only three out of 26 patients presented extension to the pylorus, but a series of observations could be made. It was noted that the tendency of extension towards the pylorus correlated with five amino acids in whole blood, namely L-Trp, L-Met, L-Lys, L-Val, and L-Leu, and five amino acids in saliva, namely D-Trp, D-Cys, D-Val, D-Ser, and D-Pip. The observations regarding the presence of an amino acid in biological fluids are presented in Table 4.

Based on the table above, an algorithm was proposed that consists of counting the amino acids that favor tumor extension to the pylorus, and it was noted that while all patients with extension to the pylorus had all five amino acids in saliva present at detectable levels, none of the patients without extension had all five amino acids present simultaneously; only 47.82% had only four amino acids present out of the five chosen. Thus, it could be said that the presence of the five amino acids in saliva is an indicator of a possible extension to the pylorus. Due to the small number of patients, our results are just an exploratory observation, and larger studies that are now ongoing in our department are needed for validation.

Regarding extension to the gastric cardia, this was present in seven of the 26 available patients, and a series of observations were noted. This extension was noted to be related to the levels of L-Gln in whole blood, L-Cys in whole blood, D-Phe in whole blood, L-Ser in whole blood, L-Pip in whole blood, L-Cys in urine, D-Ala in urine, L-Ser in urine, L-Pip in urine, L-Cys in saliva, D-Cys in saliva, D-Ala in saliva, D-Val in saliva, and D-Ser in saliva. The main characteristics are shown in Table 5.

Starting from the data in Table 5, it was observed that if only the eight amino acids that favor the presence of extension to the gastric cardia are taken into account, all patients with an extension of the tumor to the gastric cardia present at least seven of the eight amino acids with detectable levels, compared to only 36.84% of those without extension to the gastric cardia.

The last parameter analyzed is the pT value within TNM staging. This parameter complements the information provided by the tumor thickness because it evaluates the infiltration of the gastric wall layers.

According to the database, patients were classified into three groups: patients with pT1 or pT2 (13 of the 33 patients), pT3 (10 of the 33 patients), and pT4a or pT4b (10 of the 33 patients).

For the three groups, it was analyzed whether the presence or absence of an amino acid in the three biological fluids—whole blood, urine, and saliva—favors or not a certain value of the pT parameter, this being attributed to the changes that tumor cells undergo when the invasion capacity increases to the tissue destruction produced by the invasion.

After detailed analysis of the database, significant correlations were noted related to the presence or absence of D-Lys in whole blood, L-Phe in whole blood, D-Trp in urine, L-Lys in urine, D-Ala in urine, D-Trp in saliva, L-Met in saliva, L-Ala in saliva, D-Ala in saliva, D-Phe in saliva, D-Val in saliva, L-Ser in saliva, and D-Pip in saliva.

First, the selection of patients with pT4a or pT4b was followed. In this regard, Table 6 was created.

Starting from Table 6 and counting the amino acids positively correlated with the pT value, it was observed that 70% of the pT4a or pT4b patients associate the presence of at least seven of the 11 amino acids, while 65.22% of the pT1, pT2, or pT3 patients associate the presence of at most six amino acids of the 11 selected amino acids.

If the difference is made between the number of positively correlated amino acids with detectable levels in biological fluids and the number of negatively correlated amino acids with detectable levels in biological fluids, a better separation of the two categories can be made, since 70% of the pT4a or pT4b patients have a difference value of at least seven compared to only 17.4% of the pT1, pT2, or pT3 patients.

The amino acids selected in the list are not suitable for distinguishing between patients with neoplastic infiltration in the subserous adipose tissue (pT3) and those without neoplastic infiltration in the subserous adipose tissue (pT1 or pT2).

To distinguish between pT1/pT2 and pT3, it was proposed to monitor the presence of some L-amino acids in whole blood, namely L-Gln, L-Trp, L-Ala, L-Val, L-Orn, and L-Pip. Their presence was noted more frequently in pT3 patients than in pT1 or pT2. This aspect can be attributed to a greater destruction of the muscle layer with the increase in the pT value. The data supporting this observation are presented in Table 7.

These amino acids could be used to raise suspicion of invasion into the subserous adipose tissue. If amino acids with detectable levels are counted, it was observed that 90% of pT3 patients have at least four amino acids present in whole blood, while 64.29% of pT1/2 patients present at most three amino acids.

## 4. Discussion

The amino acid profile of various biological fluids has been studied previously in the context of gastric cancer. Several teams have studied the correlations between the presence of certain amino acids, their levels, and histopathological characteristics.

Gu et al. observed by monitoring plasma free amino acid levels that low levels of the amino acid Thr were associated with larger tumor size (greatest dimension > 5 cm) and deeper invasion (T ≥ 4) (*p* < 0.05) [21]. In their study, it was not mentioned whether the L, D, or both forms were measured, and it was evaluated in plasma and not in other biological fluids.

Wu et al. observed in a study in which they followed the molecular profile by mass spectrometry that L-Cys and L-Tyr were found in higher concentrations in invasive gastric cancers T3 and T4 than in T1 and T2 [22].

Their study is complementary because it evaluates amino acids by mass spectrometry at the tissue level and not in biological fluids.

The separate evaluation of the two optically active isomeric forms simultaneously in the three biological fluids provides a much broader spectrum of information on the impact that the tumor has on adjacent tissues. Also, the use of stochastic sensors that offer numerous advantages, including very low detection limits, allows a very good assessment of the presence of amino acids in biological fluids.

The amino acid profile is complex, being influenced both by nutrition and by the metabolic needs of the body and the tumor, but some assessments can be made.

L-amino acids are influenced both by nutrition, which can bring different amounts in the diet, and by the presence of the tumor, which can induce levels both low through consumption for tumor cell metabolism, and high through the occurrence of tumor necrosis and release from adjacent cells through tissue invasion.

D-amino acids occur mainly as a result of disorders in tumor cell metabolism and can be released into biological fluids both by passing into the interstitial fluid from which they reach the blood and further into saliva and urine, but they can also reach saliva through gastric regurgitation. Its presence is not dependent on nutrition, but it is supposed to be due to the torsion of DNA.

Beyond amino acids as metabolite-based biomarkers, cfDNA analysis has emerged as a complementary liquid-biopsy approach that may provide tumor-specific genomic and epigenomic information. Its integration with amino acid profiling could link systemic metabolic alterations to tumor-derived molecular changes and may improve the assessment of gastric cancer progression, thus representing a new research direction for further studies [23,24].

All included patients had values of potential confounders within normal limits, such that the body mass index ranged between 19 and 28, the samples were taken before surgery or oncological treatment, the oral microbiota was physiological, and renal function was preserved. Dietary intake was not analyzed due to a lack of information from patients. All the selected patients were negative for *Helicobacter pylori*.

## 5. Conclusions

The amino acid profile that includes both L and D stereoisomeric forms can be useful in tracking the growth, extension, and direct neoplastic infiltration in gastric cancer patients. The presence or absence of certain amino acids in relation to the detection limit of stochastic sensors, as well as their concentrations, can provide much useful information regarding tumor size and extension to the pylorus or gastric cardia in the evaluation of the pT parameter within the TNM staging.

Analysis of the molecular profile in detectable amino acids in relation to tumor location showed that tumors located in the gastric cardia and fornix are more frequently associated with the presence of L-Ser and D-Leu in whole blood and less frequently with the presence of D-Trp, D-Cys, and L-Val in saliva. Tumors located in the gastric body are more frequently associated with the presence of L-Met and D-Phe in whole blood and with D-Phe and D-Ser in saliva. Distal stomach location, in the antrum, was associated with the absence of detectable levels of the amino acids L-Gln, L-Cys, D-Met, L-Phe, D-Pip in whole blood, and L-Val in urine.

The largest dimension was positively correlated with D-Met and D-Val in urine, L-Gln and D-Gln in saliva, and negatively correlated with L-Gln and L-Ala in whole blood, with L-Met and L-Phe in urine, as well as with D-Trp, D-Phe, and D-Pip in saliva.

Tumor thickness was negatively correlated with L-Gln in whole blood, L-Ala in whole blood, L-Met in urine, D-Trp in saliva, D-Phe in saliva, and D-Pip in saliva, and positively correlated with D-Met in urine, D-Val in urine, and D-Gln in saliva.

Pyloric extension could be assessed based on D-Trp, D-Cys, D-Val, D-Ser, and D-Pip in saliva, since all patients with extension to the pylorus and duodenum were associated with the presence of all five amino acids at detectable levels in saliva.

Gastric cardiac extension was correlated with eight amino acids, namely L-Gln, L-Cys, L-Ser, and L-Pip in whole blood, L-Cys, L-Ser, L-Pip in urine, and L-Cys in saliva, noting that most patients with gastric cardiac extension to the esophagus present at least seven of the eight amino acids at detectable levels.

The degree of invasion of the gastric wall assessed by the pT value within the TNM staging system correlated with several amino acids characteristic of each value. pT4a and pT4b were positively correlated with D-Lys in whole blood, D-Trp, L-Lys in urine, and D-Trp, L-Met, L-Ala, D-Ala, D-Phe, D-Val, L-Ser, and D-Pip in saliva. The difference between pT3 and pT1/2 was based on six amino acids detectable in whole blood that occur more frequently in pT3 patients, namely L-Gln, L-Trp, L-Ala, L-Val, L-Orn, and L-Pip.

## Figures and Tables

**Table 1:** Main features of the selected patients.

Feature		Number of Patients	Percentage
Age	≥80 years	8	24.24%
	70–80 years	12	36.36%
	60–70 years	5	15.15%
	≤60 years	8	24.24%
Location	Fornix and gastric cardia	12	36.36%
	Fornix and body	2	6.06%
	Gastric body	7	21.21%
	Antrum and body	4	12.12%
	Antrum and pylorus	8	24.24%
Greatest dimension	≥6 cm	5	16.67%
	4–6 cm	14	46.67%
	<4 cm	11	36.67%
Width	≤1.5 cm	9	29.03%
	1.5–3 cm	12	38.71%
	≥3 cm	10	32.26%
Extension to pylorus	Yes	3	11.54%
	No	23	88.46%
Extension to gastric cardia	Yes	7	26.92%
	No	19	73.08%
pT value from TNM staging system	pT1/2	13	39.39%
	pT3	10	30.30%
	pT4 (a/b)	10	30.30%

**Table 2:** Main amino acids related to the greatest dimension in surface.

Amino Acid	Biological Fluid	Percentage of Patients with Detectable Levels	Nature of Correlation
L-Gln	whole blood	76.67%	Negatively correlated
L-Ala		63.33%	Negatively correlated
L-Met	urine	90.00%	Negatively correlated
D-Met		93.33%	Positively correlated
L-Phe		83.33%	Negatively correlated
D-Val		53.33%	Positively correlated
L-Gln	saliva	53.33%	Positively correlated
D-Gln		73.33%	Positively correlated
D-Trp		56.67%	Negatively correlated
D-Phe		53.33%	Negatively correlated
D-Pip		73.33%	Negatively correlated

**Table 3:** Main amino acids related to tumor width.

Amino Acid	Biological Fluid	Percentage of Patients with Detectable Levels	Nature of Correlation
L-Met	whole blood	58.06%	Negatively correlated
L-Phe		70.97%	Positively correlated
D-Val		51.61%	Negatively correlated
D-Pip		67.74%	Positively correlated
D-Trp	urine	51.61%	Positively correlated
D-Lys		77.42%	Positively correlated
L-Ala		51.61%	Negatively correlated
L-Phe		80.65%	Negatively correlated
L-Ser		51.61%	Negatively correlated
D-Ser		54.84%	Positively correlated
L-Leu		83.87%	Negatively correlated
D-Ser	saliva	64.52%	Negatively correlated

**Table 4:** Main amino acids related to the extension of the tumor to the stomach pylorus.

Amino Acid	Biological Fluid	Percentage of Patients with Extension to the Pylorus	Percentage of Patients Without Extension to the Pylorus	Nature of Correlation
L-Trp	whole blood	0.00%	21.74%	not favoring extension to the pylorus
L-Met		33.33%	69.57%	not favoring extension to the pylorus
L-Lys		0.00%	26.09%	not favoring extension to the pylorus
L-Val		66.67%	34.78%	favoring extension to the pylorus
L-Leu		33.33%	52.17%	not favoring extension to the pylorus
D-Trp	saliva	100.00%	47.83%	favoring extension to the pylorus
D-Cys		100.00%	34.78%	favoring extension to the pylorus
D-Val		100.00%	60.87%	favoring extension to the pylorus
D-Ser		100.00%	60.87%	favoring extension to the pylorus
D-Pip		100.00%	65.22%	favoring extension to the pylorus

**Table 5:** Main amino acids related to the extension of the tumor to the stomach gastric cardia.

Amino Acid	Biological Fluid	Percentage of Patients with Extension to the Gastric Cardia	Percentage of Patients Without Extension to the Gastric Cardia	Nature of Correlation
L-Gln	whole blood	100.00%	68.42%	favoring extension to the gastric cardia
L-Cys		100.00%	73.68%	favoring extension to the gastric cardia
D-Phe		71.43%	89.47%	not favoring extension to the gastric cardia
L-Ser		71.43%	57.89%	favoring extension to the gastric cardia
L- Pip		100.00%	73.68%	favoring extension to the gastric cardia
L-Cys	urine	100.00%	68.42%	favoring extension to the gastric cardia
D-Ala		28.57%	36.84%	not favoring extension to the gastric cardia
L-Ser		85.71%	42.11%	favoring extension to the gastric cardia
L- Pip		100.00%	68.42%	favoring extension to the gastric cardia
L-Cys	saliva	71.43%	63.16%	favoring extension to the gastric cardia
D-Cys		28.57%	47.37%	not favoring extension to the gastric cardia
D-Ala		28.57%	52.63%	not favoring extension to the gastric cardia
D-Val		57.14%	68.42%	not favoring extension to the gastric cardia
D-Ser		57.14%	68.42%	not favoring extension to the gastric cardia

**Table 6:** Main amino acids related to pT value from the TNM staging system.

Amino Acid	Biological Fluid	Percentage of Patients			Nature of Correlation
		pT1 and pT2	pT3	pT4a and pT4b	
D-Lys	whole blood	15.38%	20.00%	50.00%	favoring a higher pT value
L-Phe		76.92%	70.00%	60.00%	favoring a smaller pT value
D-Trp	urine	46.15%	50.00%	60.00%	favoring a higher pT value
L-Lys		30.77%	40.00%	70.00%	favoring a higher pT value
D-Ala		30.77%	30.00%	20.00%	favoring a smaller pT value
D-Trp	saliva	46.15%	60.00%	90.00%	favoring a higher pT value
L-Met		53.85%	70.00%	80.00%	favoring a higher pT value
L-Ala		53.85%	60.00%	70.00%	favoring a higher pT value
D-Ala		38.46%	50.00%	60.00%	favoring a higher pT value
D-Phe		53.85%	60.00%	70.00%	favoring a higher pT value
D-Val		53.85%	70.00%	80.00%	favoring a higher pT value
L-Ser		53.85%	60.00%	70.00%	favoring a higher pT value
D-Pip		69.23%	70.00%	90.00%	favoring a higher pT value

**Table 7:** Percentages of patients with detectable levels of the selected amino acids in pT1/2 and pT3 patients.

Amino Acid	Percentage of Patients	
	pT1 or pT2	pT3
L-Gln	69.23%	100.00%
L-Trp	23.08%	40.00%
L-Ala	46.15%	90.00%
L-Val	23.08%	70.00%
L-Orn	7.69%	40.00%
L- Pip	76.92%	100.00%

## Data Availability

Data are available from the authors upon reasonable request, subject to approval by the Ethics Committee.
